# A remarkable activity of human leukotriene A_4_ hydrolase (LTA4H) toward unnatural amino acids

**DOI:** 10.1007/s00726-014-1694-2

**Published:** 2014-02-27

**Authors:** Anna Byzia, Jesper Z. Haeggström, Guy S. Salvesen, Marcin Drag

**Affiliations:** 1 Division of Bioorganic Chemistry, Faculty of Chemistry, Wroclaw University of Technology, Wybrzeze Wyspianskiego 27, 50-370 Wrocław, Poland; 2 Division of Chemistry 2, Department of Medical Biochemistry and Biophysics, Karolinska Institutet, 171 77 Stockholm, Sweden; 3 Program in Apoptosis and Cell Death Research, Sanford Burnham Medical Research Institute, La Jolla, CA 92037 USA

**Keywords:** Aminopeptidase, Unnatural amino acid, Fluorogenic substrate, Protease, Substrate specificity

## Abstract

Leukotriene A_4_ hydrolase (LTA4H––EC 3.3.2.6) is a bifunctional zinc metalloenzyme, which processes LTA_4_ through an epoxide hydrolase activity and is also able to trim one amino acid at a time from N-terminal peptidic substrates via its aminopeptidase activity. In this report, we have utilized a library of 130 individual proteinogenic and unnatural amino acid fluorogenic substrates to determine the aminopeptidase specificity of this enzyme. We have found that the best proteinogenic amino acid recognized by LTA4H is arginine. However, we have also observed several unnatural amino acids, which were significantly better in terms of cleavage rate (*k*_cat_/*K*_m_ values). Among them, the benzyl ester of aspartic acid exhibited a *k*_cat_/*K*_m_ value that was more than two orders of magnitude higher (1.75 × 10^5^ M^−1^ s^−1^) as compared to l-Arg (1.5 × 10^3^ M^−1^ s^−1^). This information can be used for design of potent inhibitors of this enzyme, but may also suggest yet undiscovered functions or specificities of LTA4H.

**Electronic supplementary material:**

The online version of this article (doi:10.1007/s00726-014-1694-2) contains supplementary material, which is available to authorized users.

## Introduction

Leukotriene A_4_ hydrolase (LTA4H) is an enzyme with dual activities. On the one hand, it has an epoxide hydrolase activity that processes LTA_4_ into LTB_4_ to generate a chemotactic agent for human neutrophils, eosinophils, monocytes, and T cells (Haeggstrom and Funk 2011). On the other hand, LTA4H is an aminopeptidase with a catalytic zinc(II) bound to a canonical HEXXH-(X)_18_-E motif (Orning et al. 1991). The neutrophil chemoattractant proline-glycine-proline (PGP), which is a biomarker for chronic obstructive pulmonary disease and implicated in neutrophil persistence in pulmonary infections, was recently identified as an endogenous peptide substrate for LTA4H (Snelgrove et al. 2010). LTA4H can process very efficiently tripeptides with arginine at the N terminus, while dipeptidic, tetrapeptidic and pentapeptidic substrates were processed with lower efficiency by more than one order of magnitude in terms of *k*_cat_/*K*_m_ value (Orning et al. 1994). Using appropriate substrates, both the epoxide hydrolase and the aminopeptidase activity of LTA4H proceed with similar catalytic rate constants (*k*_cat_/*K*_m_ ≈ 10^6^ M^−1^ s^−1^), suggesting that both the functions of this enzyme are biologically important (Tholander et al. 2008). In addition, LTA4H can cleave, albeit much less efficiently, synthetic substrates based on single amino acids coupled with a chromophore in P1′ position, with Arg preferred over Ala (Tholander et al. 2008).

The crystal structure of LTA4H provides insight into possible substrate–enzyme interactions within the active site. It was demonstrated that tripeptides are optimal substrates for LTA4H due to their tight binding along a conserved GXMEN motif. Selectivity for Arg at the N terminus of a putative substrate was explained by a narrow S1 pocket capped with Asp-375 forming hydrogen bonds with water molecules and the guanidine group of Arg (Tholander et al. 2008). The bulk size of the S1 pocket suggests that LTA4H should recognize not only substrates with and N-terminal Arg, but also other amino acids. However, not much is known about the full aminopeptidase substrate specificity repertoire of LTA4H toward natural amino acids.

To gain insight into LTA4H preferences in recognition of substrates in the P1 pocket, we used a library of amino acids connected to a fluorophore group, 7-amino-4-carbamoylmethylcoumarin (ACC). This approach was previously used successfully to probe substrate specificity of several human, animal, plant and bacterial aminopeptidases (Poreba et al. 2012a, b; Gajda et al. 2012; Veillard et al. 2012; Zervoudi et al. 2011; Drag et al. 2010; Weglarz-Tomczak et al. 2013). In this approach, in addition to natural amino acids (proteinogenic amino acids), we also employed more than one hundred unnatural amino acids (d-amino acids, l-amino acids with different unnatural side chains). Our study uncovers a set of unexpected observations regarding the substrate specificity of LTA4H, and predicts that this enzyme may have selectivity for post-translationally modified N-terminal amino acids.

## Materials and methods

### General

All chemicals were obtained from commercial sources and used without further purification. Rink amide (aminomethyl) polystyrene (100–200 mesh, extent of labeling 0.46 mmol g^−1^ N^−1^ loading, 1 % cross-linked), piperidine and trifluoroacetic acid (TFA) were from Iris Biotech GmbH, Fmoc protected amino acids were from Iris Biotech GmbH, Sigma Aldrich, Fluka and Novabiochem. 1-Hydroxybenzotriazole hydrate (HOBt), *O*-(7-azabenzotriazol-1-yl)-*N,N,N′N*′-tetramethyluronium hexafluorophosphonate (HATU), *N,N*-diisopropylethylamine (DIPEA), and triisopropylsilane (TIPS) were purchased from Sigma Aldrich. 7-Fmoc-aminocoumarin-4-acetic acid (Fmoc-ACC) was synthesized according the procedure by (Maly et al. 2002). LC–MS data were recorded with the Shimadzu LCMS-2010EV system at the and the Department of Biotechnology, University of Wroclaw. Analytical high performance liquid chromatography (HPLC) analysis was performed with a Waters M600 solvent delivery module equipped with a Waters M2489 detector system using preparative Waters Spherisorb S10ODS2 or analytical Waters Spherisorb S5ODS2 columns. Preparative HPLC was carried out with a Waters M600 solvent delivery module equipped with a Waters M2489 Detector system and a C18 column. Solvent composition: system A: water/0.1 % TFA and system B: acetonitrile 80 %/water 20 % with 0.1 % TFA. To determine turnover rates of individual substrates a Spectra MAX Gemini EM fluorimeter (Molecular Devices) operating in the kinetic mode in 96-well plates was used. The excitation/emission wavelengths were established at 355 nm/460 nm.

### Enzyme isolation

Human recombinant LTA4H was expressed in *E. coli* and purified to apparent homogeneity as previously described (Rudberg et al. 2002).

### Library synthesis

An ACC fluorophore with Fmoc protecting group (2.5 eq.) was coupled to deprotected Rink Amide Resin (1 eq.) using Hydroxybenzotriazole hydrate (HOBt––2 eq.) and HBTU as coupling reagent in dimethylformamide (DMF) solvent for 24 h. To increase the level of ACC substitution to the resin, this procedure was repeated one more time with half amount of reagents. Next, the protecting group was removed by 20 % of piperidine/DMF for two times 5 min, and once 20 min. The resin was washed with DMF (about 5–6 times) and three times with dichloromethane and three times with methanol, and left in vacuum for 24 h to be dried. The resin was divided equally into separate wells of 48-well cartridge and swelled with dichloromethane for 2 h, then washed with DMF three times. The coupling reaction of Fmoc-protected amino acids (2.5 eq.) to ACC was carried out in the presence of HATU (2.5 eq.) and 2,4,6-collidine (2.5 eq.) in DMF for 24 h. Also here, to increase the level of amino acid substitution to the resin, this procedure was repeated one more time with half amount of reagents. Next, the resin was washed five times with DMF and the protecting group was removed by 20 % of piperidine/DMF for 5, 5 and 20 min. The resin was washed with DMF (about 5–6 times), three times with dichloromethane and three times with methanol, and left in vacuum for 24 h. Substrate cleavage from the resin was performed in cold 95 % TFA, 2.5 % H_2_O, 2.5 % triisopropyl silane for 1.5 h. Finally, the substrates were precipitated in diethyl ether for 30 min at 4 °C and centrifuged. Obtained substrates were purified and analyzed using HPLC. After lyophilization compounds were analyzed by mass spectrometry and dissolved in anhydrous DMSO to a final concentration of 50 mM.

### Substrate library assay

A library of 130 amino acids coupled to the ACC fluorophore was used to screen the substrate specificity of LTA4H. It consists of 19 of the 20 natural amino acids (not cysteine, which is prone to oxidation), 18 d-amino acids, and the others are l-derivatives of unnatural amino acids. The enzyme was assayed in 250 mM tris buffer, pH 7.5, containing 500 mM KCl and 0.1 % of bovine serum albumin, both of which stimulate the aminopeptidase activity (Wetterholm and Haeggstrom 1992; Orning and Fitzpatrick 1992). The buffer was prepared at room temperature. Enzyme was incubated for 30 min at 37 °C and added to the substrates in the wells of a 96-well plate. The fluorescence increase (Relative Fluorescence Unit per second) was monitored using Spectra MAX Gemini EM fluorimeter operating in kinetic mode (Molecular Devices, the excitation wavelength was 355 nm and emission was 460 nm). The time of each assay was 30 min, but only the linear portion of the curve was used to calculate velocity. The final enzyme concentration was in the range of 30–40 nM. Individual substrate concentrations were 2 μM, which is low enough below estimated *K*_M_ values to obtain velocity values proportional to the initial velocity for each substrate. The DMSO concentration in each assay was below 2 %. Enzymatic hydrolysis of each substrate was measured at least three times. Results are presented as relative activity of each substrate (the efficiency of fluorophore release) in comparison to the best one (100 %).

### Determination of kinetic parameters for best substrates (*k*_cat_, *K*_m_, *k*_cat_/*K*_m_)

The best substrates were selected to determine kinetic parameters of hydrolysis by LTA4H. The assay condition was the same as described above. The ACC concentration and corresponding Relative Fluorescence Unit was calculated by total hydrolysis of five independent substrates at the same concentration. The average value was used for further calculations. To determine kinetic parameters, velocities for eight different concentrations of each substrate were measured. Each experiment was repeated at least three times and results are presented as mean ± SD. The enzyme concentration was 30 nM and the substrate concentrations for *k*_cat_/*K*_m_ determination ranged from 1 to 500 μM. The total time for each experiment was between 15 and 30 min and only the linear portions of the curves were used for calculations. For natural amino acids, with exception of l-Arg, activity was low and did not obey saturation kinetics within the substrate range tested, suggesting increased *K*_m_ and reduced *k*_cat_. Only a rough estimation of *k*_cat_/*K*_m_ was possible (Table 1). The final DMSO concentration was below 2 %.

**Table 1:** Comparison of the catalytic efficiency (*k*_cat_/*K*_m_) of the selected natural amino acid based substrates

Natural amino acid	*k*_cat_/*K*_m_ (M^−1^ s^−1^)
Ala	470.6 ± 91.5
Arg	1,496.7 ± 102.8
Leu	343.4 ± 57.7
Lys	617.4 ± 80.1
Met	297.8 ± 92.3
Phe	747.4 ± 104.9
Pro	449.1 ± 41.9

## Results

### The substrate library

To determine the ability of LTA4H to recognize and hydrolyze different substrates we used a library of natural and unnatural amino acids coupled to the ACC fluorophore used in previous studies (Drag et al. 2010; Zervoudi et al. 2011). In addition, we extended this library by several new derivatives of unnatural amino acids using a previously described methodology (Drag et al. 2010; Zervoudi et al. 2011). The library consists of compounds with side chains of unnatural amino acids characterized by significant structural diversity. Small or bulky, branched or unbranched, acidic, neutral or basic, hydrophobic or hydrophilic side chains guarantee detailed probing of the S1 pocket of LTA4H. All structures and information about fluorogenic substrates are in Online Resource 1. To verify the requirements of LTA4H for substrates with unblocked α-amino group (a vital feature for aminopeptidases) we checked the ability of this enzyme to recognize substrates with secondary amine derivatives (Pro, Tic, Oic) and with modified main chain (Apns (2S,3S), β-Ala, 6-Ahx). Stereoselectivity of the enzyme was investigated with d-derivatives of selected amino acids.

### Substrate specificity screening assay

To establish the optimal condition for LTA4H catalysis we performed a preliminary screen of the library. The best substrate containing a natural amino acid, l-Arg-ACC, had a *K*_m_ value of 301 μM, whereas the lowest measured *K*_m_ value was for the substrate l-Bpa-ACC with an unnatural amino acid was 2.55 μM. Subsequently, to evaluate substrate specificity at conditions where substrate cleavage velocities are proportional to *k*_cat_/*K*_m_, we performed screening at a final substrate concentration of 1 μM, which was more than two times lower than the lowest established *K*_m_ value. To validate this approach, we performed an additional screen at substrate concentrations of 5 μM. This did not affect the observed overall data, revealing that the *K*_m_ values for these substrates were higher than 5 μM, thus guaranteeing a proportional correlation between fluorescence signal and *k*_cat_/*K*_m_.

### Specificity toward natural and d-amino acids

**Fig. 1:**
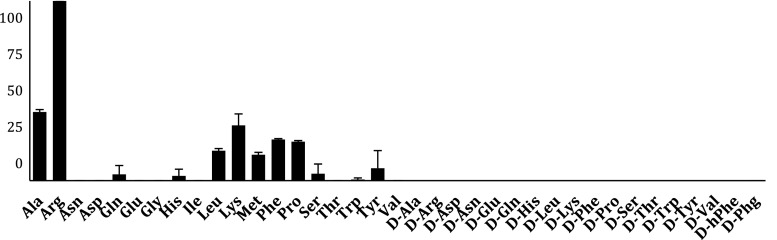
Preferred natural amino acid substrates for LTA4H. Initial screening of the 19-membered natural amino acid substrate library and 18 d-amino acid substrates. Enzyme activity was monitored using an fMax multi-well fluorescence plate reader (Molecular Devices) at excitation wavelength of 355 nm and an emission wavelength of 460 nm. The *x-axis* represents the abbreviated amino acid names (for full name and structure see Fig. S1). The *y-axis* represents the average relative activity expressed as a percent of the best amino acid substrate. All structures and information about fluorogenic substrates are in Online Resource 1

The best substrate for LTA4H with a natural amino acid was l-Arg, which was cleaved more than two times more rapidly compared to the second best natural amino acid l-Ala (Fig. 1). Preferred substrates generally had hydrophobic or basic properties, and negligible hydrolysis of l-Gln, l-His, l-Ser and l-Trp was observed. Extremely high stereospecificity of LTA4H in the S1 pocket was demonstrated by lack of processing of d-amino acids.

Subsequent calculation of *k*_cat_/*K*_m_ parameters for the best seven natural amino acids matched very well with library screening data. The highest observed *k*_cat_/*K*_m_ for l-Arg was 1,496.7 M^−1^ s^−1^, while l-Phe, the next well-cleaved substrate was about two times lower (Table 1). Other amino acids were processed by LTA4H proportionally lower. Interestingly, l-Pro is quite efficiently hydrolyzed by LTA4H, while in previous studies this substrate was not recognized by aminopeptidase N (CD13), which belongs to the same family (Drag et al. 2010; Tholander et al. 2008).

### Specificity toward substrates containing unnatural amino acids

**Fig. 2:**
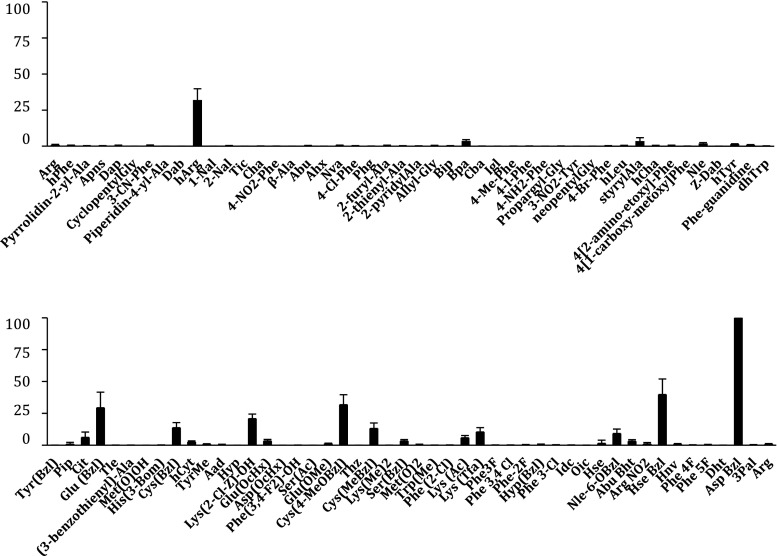
Individual preferences in the S1 pocket of LTA4H toward unnatural amino acid substrates compared to the best natural amino acid conjugate, l-Arg. Enzyme activity was monitored using an fmax multi-well fluorescence plate reader (molecular devices) at excitation wavelength of 355 nm and an emission wavelength of 460 nm. The *x-axis* represents the abbreviated amino acid names. The *y-axis* represents the average relative activity expressed as a percent of the best amino acid. All structures and information about fluorogenic substrates are in Online Resource 1

Interestingly, LTA4H demonstrated enhanced activity toward a significant number of substrates containing unnatural amino acids. The most striking preference was observed for *O*-benzyl esters of aspartic acid (AspBzl) and for homoarginine (hArg) (Fig. 2). The most preferred amino acid conjugates were processed by LTA4H at rates never observed before in direct comparison of natural and unnatural amino acid preferences by previously investigated aminopeptidases. One common feature of all the preferred unnatural amino acid substrates is a very large, long, non-charged and hydrophobic character of the side chain, demonstrating that the S1 pocket of this enzyme is very spacious and able to accommodate much bigger moieties compared to the biggest natural amino acids.

To gain better insight into the unusual activity of LTA4H toward unnatural amino acid substrates, we measured detailed kinetic parameters (*k*_cat_, *K*_m_, and *k*_cat_/*K*_m_) for the nine best (Table 2). The obtained data confirmed the results from the library screening. The highest catalytic efficiency was observed for l-AspBzl (*k*_cat_/*K*_m_ = 1.75 × 10^5^ M^−1^ s^−1^), which was more than one hundred times higher than the value obtained for the best natural amino acid conjugate, l-Arg. The catalytic efficiencies of the other substrates matched well the rates obtained during library screening. Interestingly, extension of the alkyl chain of l-Arg by one methylene group to l-hArg dramatically improved the processing of this substrate by LTA4H.

**Table 2:**
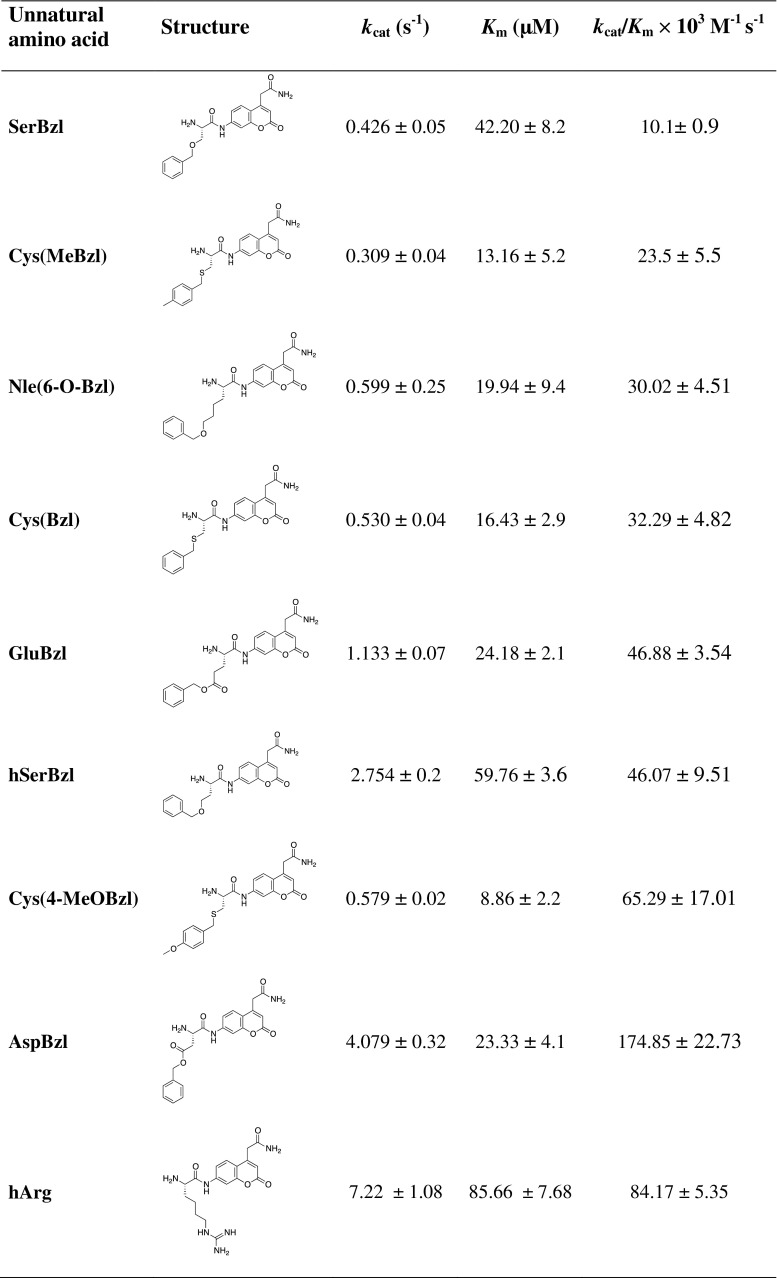
Comparison of the catalytic efficiency (*k*_cat_/*K*_m_) of selected unnatural amino acid based substrates

Similarly to other previously tested aminopeptidases, LTA4H had a very strong preference for amino acids with an amino group in the α position and was inactive toward substrates with a hydroxyl group instead of an amine (Apns) or amino acids without amino groups present in this position (6-Ahx or β-Ala) (Drag et al. 2010; Zervoudi et al. 2011). This confirms that the aminopeptidase activity of LTA4H is consistent with other enzymes from this family.

Finally, LTA4H was incubated with a range of previously studied general aminopeptidase phosphonate inhibitors (Arg-PO_3_H_2_, Nle-PO_3_H_2_ and hPhe-PO_3_H_2_) at concentration of 200 μM for 30 min (Drag et al. 2010; Kannan Sivaraman et al. 2013; Weglarz-Tomczak et al. 2013). No cleavage of the library substrates in the presence of these compounds confirms that the observed fluorescence signal in incubations without inhibitors is the result of LTA4H aminopeptidase activity.

## Discussion

Leukotriene A_4_ hydrolase/aminopeptidase is a bifunctional zinc metalloenzyme that recently attracted considerable attention due to its crucial role in limiting chronic pulmonary neutrophilic inflammation (Snelgrove et al. 2010). However, despite a recent strong interest in development of LTA4H inhibitors, the detailed substrate specificity of this enzyme had not previously been investigated. Especially, intriguing was the fact that the structures published so far of inhibitors docked to this enzyme reveal a spacious S1 pocket, suggesting that the size of this pocket is large enough to accept molecules that are much bulkier than l-Arg, the natural amino acid best recognized by LTA4H (Thunnissen et al. 2001). To test this hypothesis, we systematically investigated the substrate specificity of this enzyme using a large library of fluorogenic substrates. The results that we obtained strongly confirmed our hypothesis.

The best substrates in our study were derivatives of natural amino acids, l-Asp, l-Glu, l-Cys and l-Ser that were protected with bulky and spacious groups. Interestingly, these amino acids (cysteine was not tested) in their unprotected native form were poorly cleaved (l-Ser) or not cleaved at all (l-Asp and l-Glu) by LTA4H. A separate example here is l-hArg, which is only one methylene group longer than the proteinogenic amino acid l-Arg, but is processed by LTA4H at a markedly higher rate. A likely explanation to this may be the electronegative nature of the bottom of the S1 pocket, with the dominant Asp-375 forming hydrogen bonds with l-Arg, as demonstrated in an analysis of the crystal structure of LTA4H (Tholander et al. 2008). Most likely, elongation of the alkyl chain of l-Arg results in even better alignment of the l-hArg substrate and thus much more efficient hydrolysis.

Our studies also demonstrate that substrates based on very bulky and particularly hydrophobic unnatural amino acids such as l-hPhe, l-hCha or l-Bip were cleaved by LTA4H albeit at a significantly lower rate compared to the best substrates identified in our study. This suggests that the enzyme prefers very bulky substrates, which in structure possesses hydrophilic atom(s) like oxygen, sulfur or nitrogen, to achieve optimal binding in the S1 pocket. An example that confirms this conclusion is l-Bpa, whose side chain has two hydrophobic phenyl rings and is quite similar to l-Bip, but has an additional oxygen atom in the side chain. As a result, l-Bpa is much more efficiently cleaved compared to l-Bip. Recent studies with β-naphthylamide substrates demonstrated also an advantage of l-Bpa over l-Bip (Poras et al. 2013). On the other hand, benzyl protected tyrosine is quite weakly processed by LTA4H. It seems that despite the presence of a big hydrophobic moiety and oxygen in the side chain structure, this derivative is already very big and demonstrates space limitations within the S1 pocket. Interestingly, despite a high similarity with the best cleaved l-AspBzl, cyclohexyl esters of aspartic acid (l-Asp(OcHx) or glutamic acid (l-Glu(OcHx) are hydrolyzed at significantly lower rates by LTA4H. This suggests that for optimal binding in the S1 pocket a hydrophobic group is also required, but of aromatic character, like a phenyl ring present in the best processed substrates in our study. Analysis of previously published crystal structures of LTA4H in complex with inhibitors confirms our data. For example, (Kirkland et al. 2008) synthesized a series of arylamide derivatives of glutamic acid, which were potent inhibitors of LTA4H. These compounds were based on glutamic acid, the side chain of which was protected with a hydrophobic phenyl ring derivatized with different substituents. In another study, (Enomoto et al. 2009) obtained *S*-(4-cyclohexyl)benzyl-cysteine derivatives as potent and selective LTA4H inhibitors, which contained a sulfhydryl group of cysteine protected with very bulky and hydrophobic phenyl and additional cyclohexyl rings. Moreover, a recent comprehensive review on LTA4H inhibitors demonstrates several structures with features in common with the above discussed structural elements preferred in the S1 pocket of LTA4H (Caliskan and Banoglu 2013). Finally, analysis of the crystal structure of LTA4H demonstrates that besides the electronegative properties at the bottom of the S1 pocket, its upper parts are more neutral and facilitate binding of hydrophobic groups (Tholander et al. 2008).

In addition to providing information about substrate specificity, a very intriguing observation in our study was the dramatic increase of the catalytic efficiency of substrates based on unnatural amino acids compared to those containing natural ones. Usually, in our previous studies, the maximal increase in efficiency was about 2–3-fold (Drag et al. 2010; Poreba et al. 2012b; Zervoudi et al. 2011). To the best of our knowledge, a gain in catalytic efficiency exceeding two orders of magnitude has never been observed with synthetic substrates for any type of exopeptidase. One could perhaps expect this result with tri- or tetrapeptidic substrates for endoproteases, but with conjugates of single amino acids for exopeptidases this result is highly unusual. It provides evidence for the potential of unnatural amino acids to be applied in substrate activity screenings of proteolytic enzymes, but also raises the question of the molecular basis for this effect. Recently, we speculated that substrate specificity of proteases could be dictated by posttranslational modifications, an issue that has not been investigated in detail before (Kasperkiewicz et al. 2012). Our observation of increased catalytic efficiency against side-chain modified versions of l-Asp, l-Glu, l-Cys and l-Ser seems to fit perfectly with this scenario. However, it is difficult to speculate about the nature of putative natural amino acid modifications based on data with only single amino acids and this issue will certainly require further studies, most probably using proteomic methods on endogenous substrates.

Our study also confirms the unusual LTA4H activity toward substrates containing l-Pro. The secondary amine at its N terminus is normally not well recognized by enzymes of the M1 family of metallopeptidases with the GXMEN motif, which is responsible for interactions with the free amine in α position (Tholander et al. 2008). However, processing of l-Pro substrates in our studies is consistent with the ability of LTA4H to hydrolyze the endogenous substrate Proline-Glycine-Proline (PGP), a chemoattractant for leukocytes (Snelgrove et al. 2010).

In summary, we have performed a detailed analysis of the S1 pocket specificity of LTA4H aminopeptidase. Using substrate library screening we demonstrated key features of this enzyme, disclosing particular preferences in shape and size of potential substrates and inhibitors, which can facilitate rational design of small molecular-mass selective markers that can be used in studies of LTA4H.

## Electronic supplementary material

Below is the link to the electronic supplementary material.

Supplementary material 1 (DOCX 1,250 kb)
