# Supplementary material for: A remarkable activity of human leukotriene A4 hydrolase (LTA4H) toward unnatural amino acids
Source: Amino Acids. 2014 Feb 27;46(5):1313–20. doi: 10.1007/s00726-014-1694-2 (PMC3984412; doi:10.1007/s00726-014-1694-2)
Supplement: Supplementary file 1 — Supplementary material 1 (DOCX 1,250 kb) [file 726_2014_1694_MOESM1_ESM.docx]

**A remarkable activity of human leukotriene A_4_ hydrolase (LTA4H) toward unnatural amino acids**

Anna Byzia^1^, Jesper Z. Haeggström^2^, Guy S. Salvesen^3^, Marcin Drag^1^

*^1^Division of Bioorganic Chemistry, Wroclaw University of Technology, Wybrzeze Wyspianskiego 27, 50-370 Wroclaw, Poland; ^2^Division of Chemistry 2, Department of Medical Biochemistry and Biophysics, Karolinska Institutet, S-171 77 Stockholm, Sweden; ^3^Program in Apoptosis and Cell Death Research, Sanford Burnham Medical Research Institute, La Jolla, CA 92037, USA*

Address correspondence to: Marcin Drag, Ph.D., Division of Bioorganic Chemistry, Faculty of Chemistry, Wroclaw University of Technology, 50-370 Wroclaw, Poland;

E-mail: [marcin.drag@pwr.wroc.pl](mailto:marcin.drag@pwr.wroc.pl); phone: +48 71 320 4526; fax: +48 71 320 2427

**Electronic Supplementary Material**

**Table ESP1.** Structures, names and abbreviations of all amino acid fluorogenic substrates used in the studies.

| ENTRY | Name |  | STRUCTURE | M.W. |
| --- | --- | --- | --- | --- |
| 1 | L-Alanine-ACC | Ala | 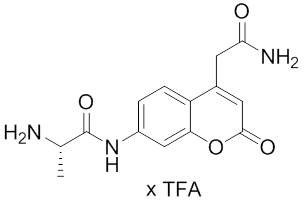 | 289,1 |
| 2 | L-Arginine-ACC | Arg | 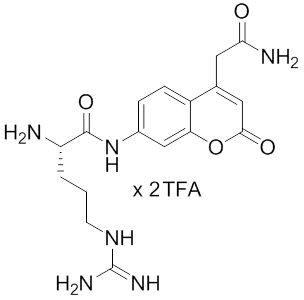 | 376,2 |
| 3 | L-Asparagine-ACC | Asn | 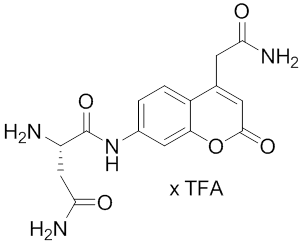 | 332,1 |
| 4 | L-Aspartic acid-ACC | Asp | 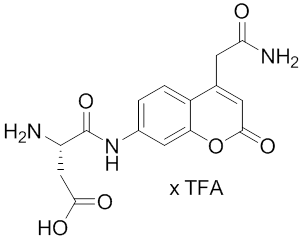 | 333,1 |
| 5 | L-Glutamine-ACC | Gln | 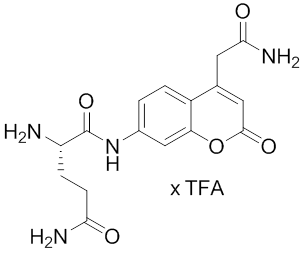 | 346,2 |
| 6 | L-Glutamic acid-ACC | Glu | 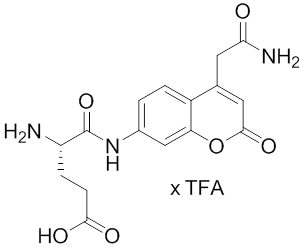 | 347,1 |
| 7 | Glycine-ACC | Gly | 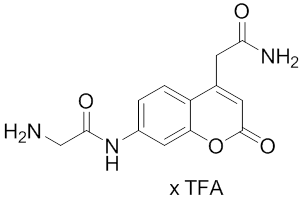 | 275,1 |
| 8 | L-Histidine-ACC | His | 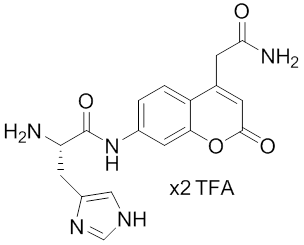 | 355,2 |
| 9 | L-Isoleucine-ACC | Ile | 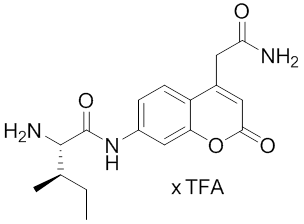 | 331,2 |
| 10 | L-Leucine-ACC | Leu | 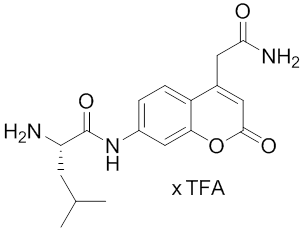 | 331,2 |
| 11 | L-Lysine-ACC | Lys | 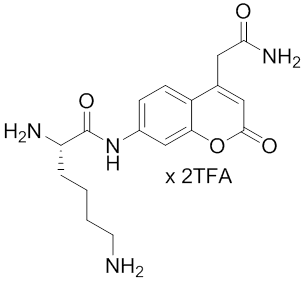 | 346,2 |
| 12 | L-Methionine-ACC | Met |  | 349,1 |
| 13 | L-Phenylalanine-ACC | Phe | 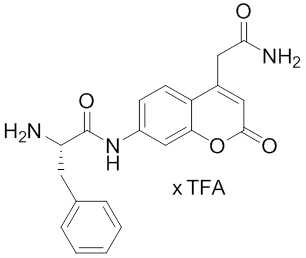 | 365,2 |
| 14 | L-Proline-ACC | Pro | 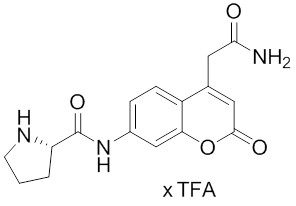 | 315,1 |
| 15 | L-Serine-ACC | Ser | 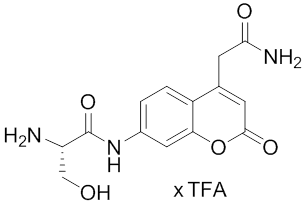 | 305,1 |
| 16 | L-Threonine-ACC | Thr | 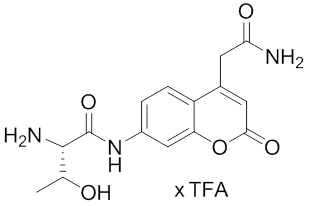 | 319,1 |
| 17 | L-Trypthophan-ACC | Trp | 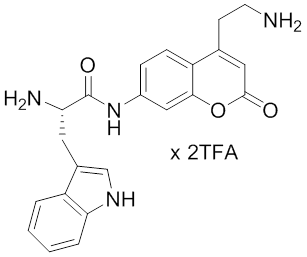 | 404,2 |
| 18 | L-Tyrosine-ACC | Tyr | 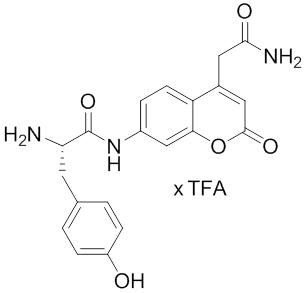 | 381,2 |
| 19 | L-Valine-ACC | Val | 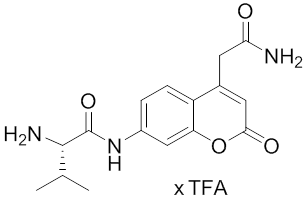 | 317,2 |
| 20 | L-homoPhenylalanine-ACC | hPhe | 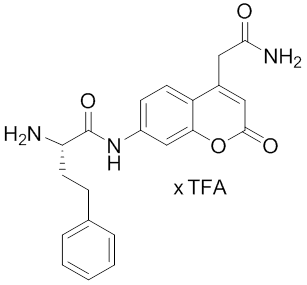 | 379,2 |
| 21 | (1-pyrrolidin-2-yl)-L-Alanine-ACC | (1-pyrrolidin-2-yl)-Ala | 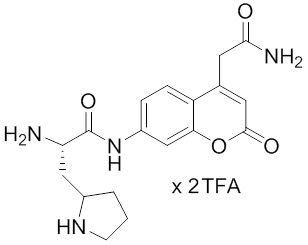 | 358,2 |
| 22 | (2S,3S)-3-amino-2-hydroxy-4-phenyl-buturic acid-ACC | Apns (2S, 3S) | 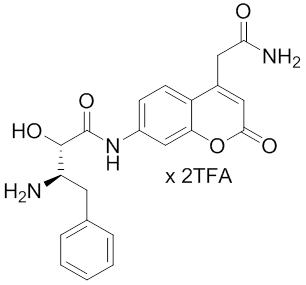 | 395,2 |
| 23 | 2,3-diaminopropionic acid-ACC | Dap | 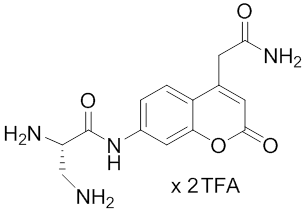 | 304,1 |
| 24 | cyclopentyl-L-Glycine-ACC | cyclopentyl-Gly | 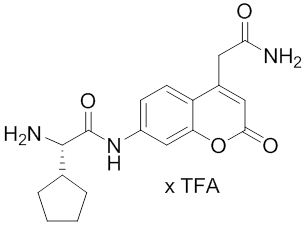 | 343,2 |
| 25 | 4-cyano-L-Phenylalanine-ACC | 3-CN-Phe | 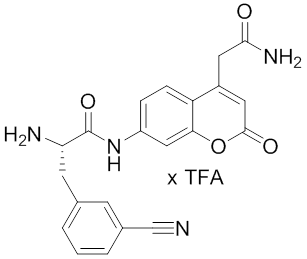 | 390,2 |
| 26 | (1-piperidin-4yl)-L-Alanine-ACC | (1-piperidin-4yl)-Ala | 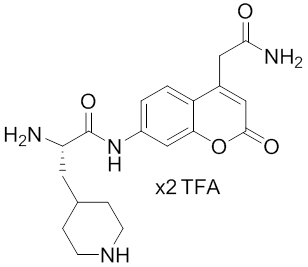 | 372,2 |
| 27 | L-2,4-diaminobutyric acid-ACC | Dab | 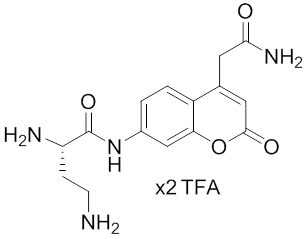 | 318,2 |
| 28 | L-homoArginine-ACC | hArg | 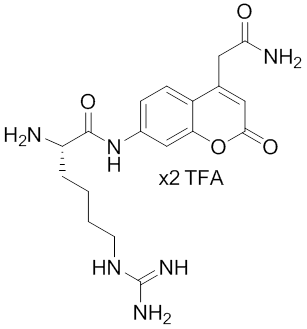 | 388,2 |
| 29 | (1-naphthyl)-L-Alanine-ACC | 1-NaI | 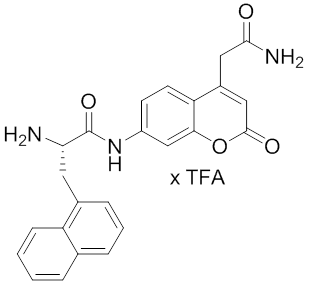 | 415,2 |
| 30 | (2-naphthyl)-L-Alanine-ACC | 2-NaI | 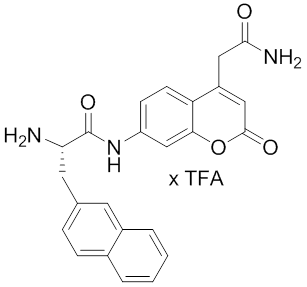 | 415,2 |
| 31 | L-1,2,3,4-tetrahydroisoquinoline-3-carboxylic acid-ACC | Tic | 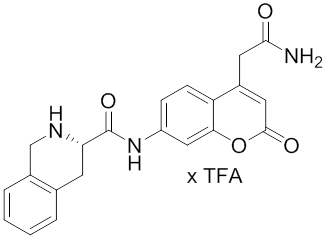 | 377,2 |
| 32 | cyclohexyl-L-Alanine-ACC | Cha | 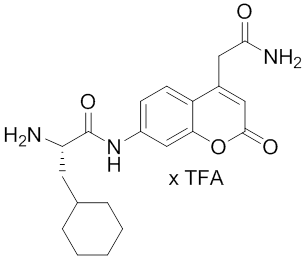 | 371,2 |
| 33 | 4-nitro-L-Phenylalanine | 4-NO_2_-Phe | 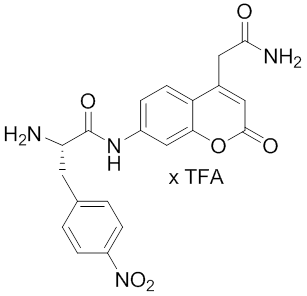 | 410,1 |
| 34 | beta-Alanine | β-Ala | 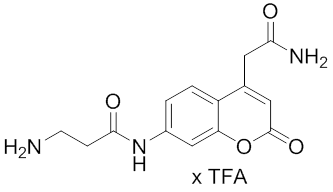 | 289,1 |
| 35 | L-aminobutyric acid-ACC | Abu | 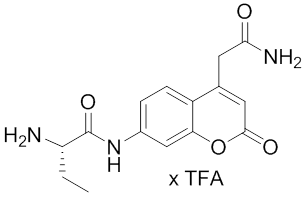 | 303,1 |
| 36 | 6-(amino)hexanoic acid-ACC | 6-Ahx | 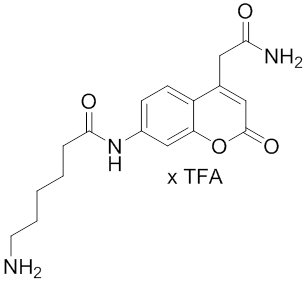 | 331,2 |
| 37 | L-norValine-ACC | Nva | 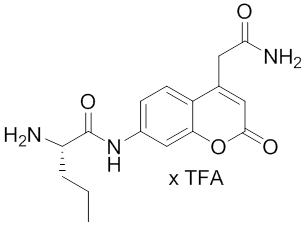 | 317,2 |
| 38 | 4-chloro-L-Phenylalanine-ACC | 4-Cl-Phe | 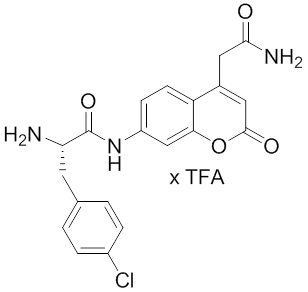 | 399,1 |
| 39 | L-α-phenyl-Glycine-ACC | Phg | 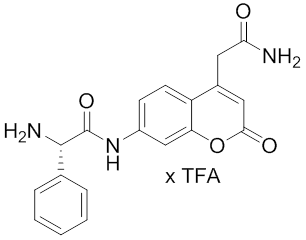 | 351,1 |
| 40 | 2-furyl-L-Alanine-ACC | 2-furyl-Ala | 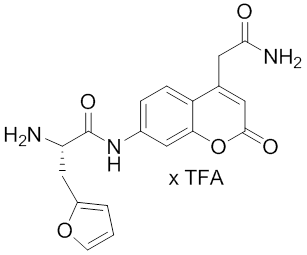 | 355,1 |
| 41 | 2-thienyl-L-Alanine-ACC | 2-thienyl-Ala | 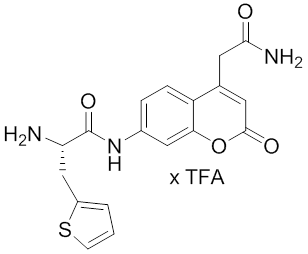 | 371,1 |
| 42 | 2-pyridyl-L-Alanine-ACC | 2-pyridyl-Ala | 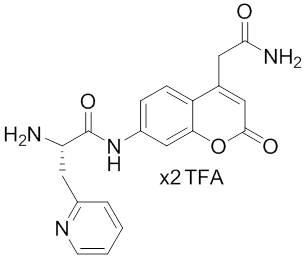 | 366,1 |
| 43 | L-allyl-Glycine-ACC | allyl-Gly | 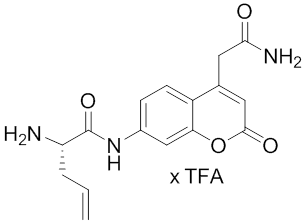 | 315,1 |
| 44 | biphenyl-L-Alanine-ACC | Bip | 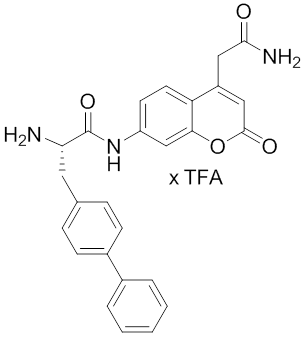 | 441,2 |
| 45 | 4-benzoyl-L-Phenylalanine-ACC | Bpa | 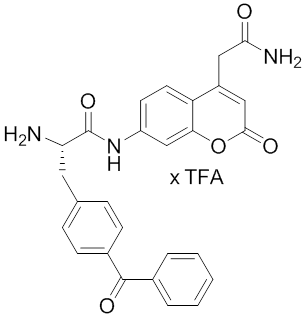 | 469,2 |
| 46 | L-2-Amino-4-cyanobutyric acid-ACC | Cba | 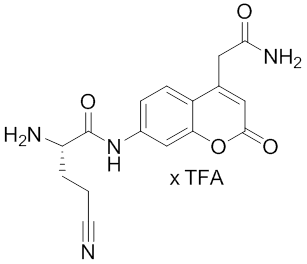 | 328,1 |
| 47 | L-2-Indany-Glycine-ACC | Igl | 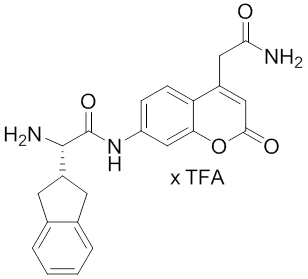 | 391,2 |
| 48 | 4-methyl-L-Phenylalanine-ACC | 4-Me-Phe | 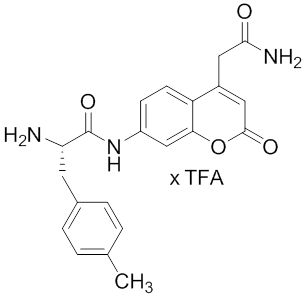 | 379,2 |
| 49 | 4-iodo-L-Phenylalanine-ACC | 4-I-Phe |  | 491,0 |
| 50 | 4-amino-L-Phenylalanine | 4-NH_2_-Phe | 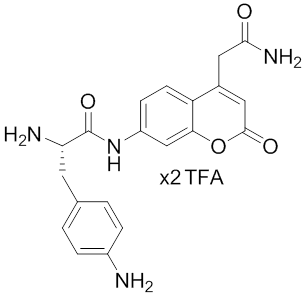 | 380,1 |
| 51 | L-propargyl-Glycine-ACC | propargyl-Gly | 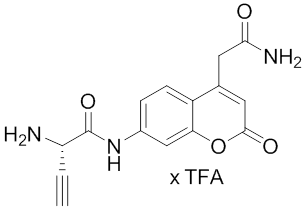 | 313,1 |
| 52 | 3-nitro-L-Tyrosine-ACC | 3-NO_2_-Tyr | 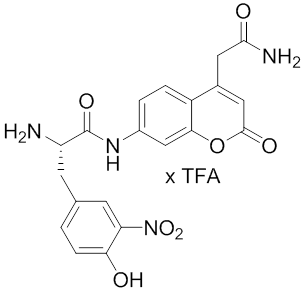 | 426,1 |
| 53 | L-neopentyl-Glycine-ACC | neopentyl-Gly | 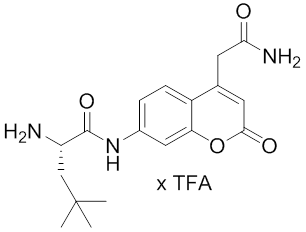 | 345,2 |
| 54 | 4-bromo-L-Phenylalanine-ACC | 4-Br-Phe | 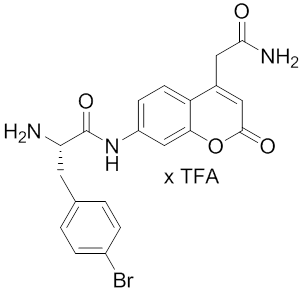 | 443,1 |
| 55 | L-homoLeucine-ACC | hLeu | 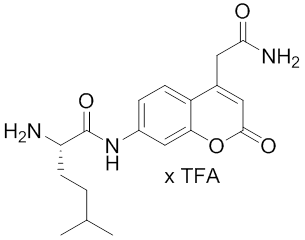 | 345,2 |
| 56 | styryl-L-Alanine-ACC | styryl-Ala | 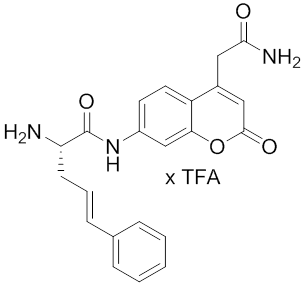 | 391,2 |
| 57 | L-homocyclohexyl Alanine-ACC | hCha | 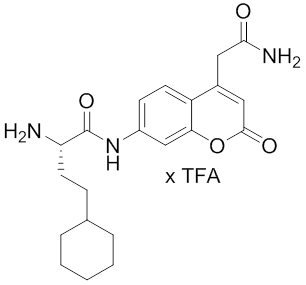 | 385,2 |
| 58 | 4[2-(amino)-ethoxy]-L-Phenylalanine-ACC | 4[2-(amino)-ethoxy]-Phe | 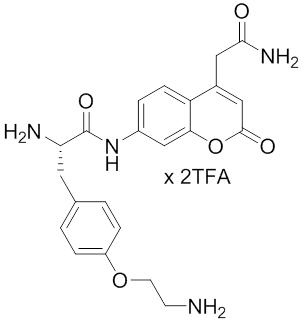 | 424,2 |
| 59 | 4[1-(carboxy)-methoxy]-L-Phenylalanine-ACC | 4[1-(carboxy)-methoxy]-Phe | 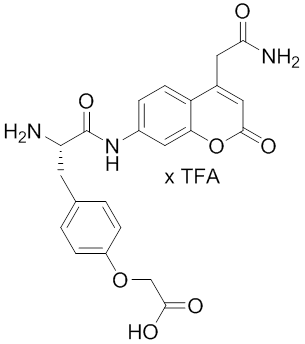 | 439,2 |
| 60 | L-norLeucine-ACC | Nle | 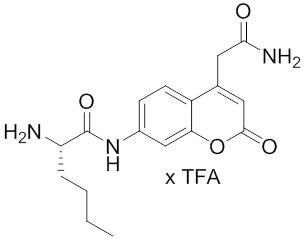 | 331,2 |
| 61 | β-Z-L-3,4-diaminobutyric acid-ACC | b-Z-Dab | 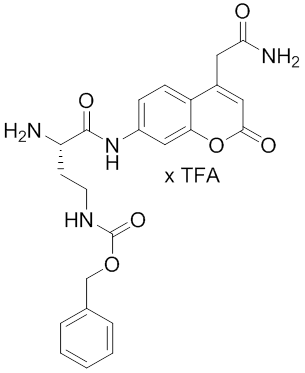 | 452,2 |
| 62 | L-homoTyrosine-ACC | hTyr | 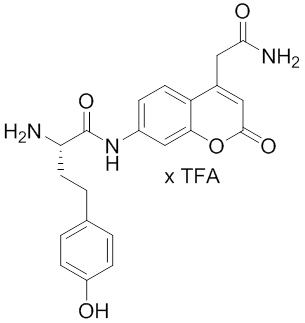 | 395,4 |
| 63 | guanidine-L-Phenylalanine-ACC | Phe-guanidine | 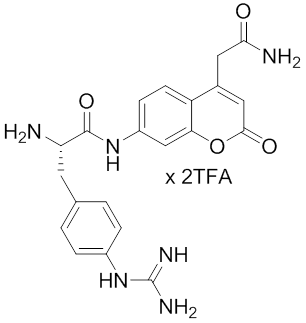 | 422,4 |
| 64 | L-Dihydro-Trypthophan | dhTrp | 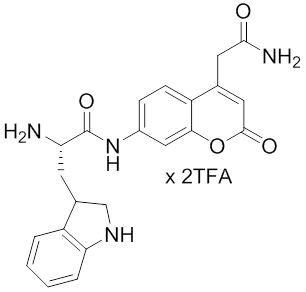 | 406,4 |
| 65 | benzyl-L-Tyrosine-ACC | Tyr(Bzl) | 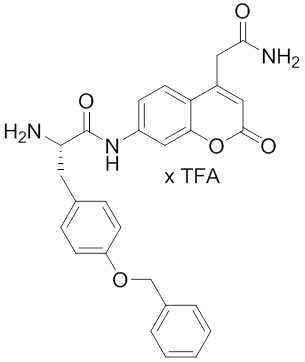 | 471,2 |
| 66 | piperidine-2-carboxylic acid-ACC | Pip | 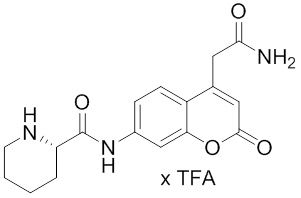 | 329,1 |
| 67 | L-Citruline-ACC | Cit | 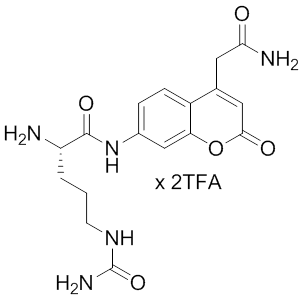 | 375,2 |
| 68 | L-Glutamic acid 5-benzyl ester -ACC | Glu(Bzl) | 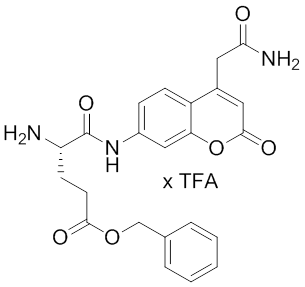 | 437,2 |
| 69 | L-tert-Leucine-ACC | Tle | 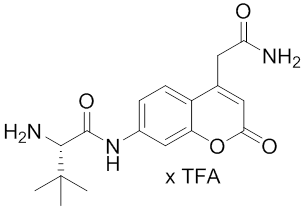 | 331,2 |
| 70 | (3-benzothienyl)-L-Alanine-ACC | (3-benzothienyl)-Ala | 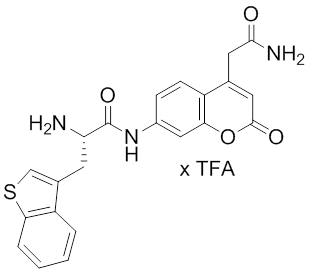 | 421,4 |
| 71 | L-Methionine sulfoxide-ACC | Met(O)OH | 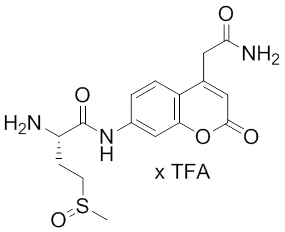 | 365,4 |
| 72 | *N(im)*-benzyloxymethyl-L-Histidine-ACC | His(3-Bom) | 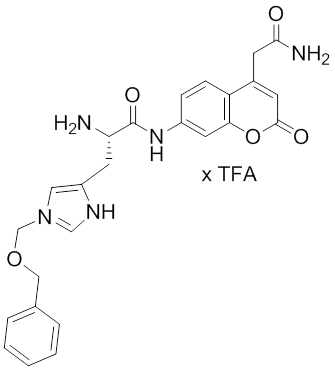 | 476,5 |
| 73 | (S)-Benzyl-L-Cysteine-ACC | Cys(Bzl) | 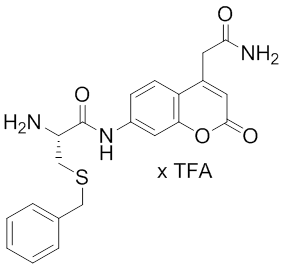 | 411,2 |
| 74 | L-homoCitruline-ACC | hCit | 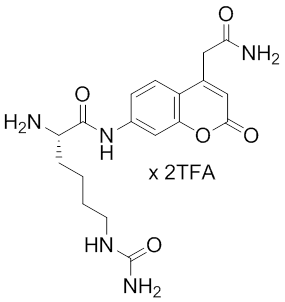 | 389,4 |
| 75 | *O*-methyl-L-Tyrosine-ACC | TyrMe | 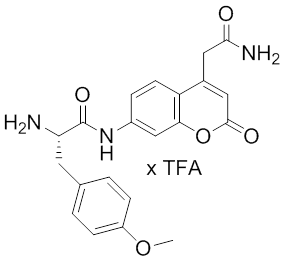 | 395,4 |
| 76 | L-homoGlutamic acid-ACC | Aad | 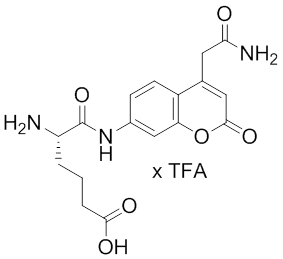 | 361,4 |
| 77 | L-hydroxyProline-ACC | Hyp | 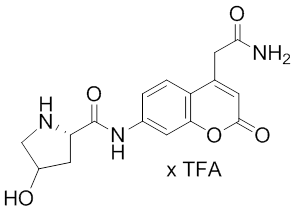 | 331,3 |
| 78 | N-(2-chlorobenzyloxycarbonyl)-L-Lysine-ACC | Lys(2-Cl-Z)-OH | 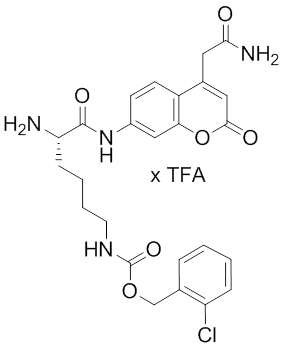 | 515,0 |
| 79 | L-Glutamic acid 5-cyclohexyl ester-ACC | Glu(OcHx) | 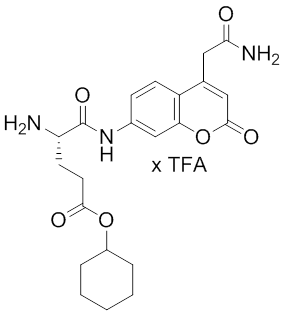 | 429,1 |
| 80 | L-Aspartic acid 5-cyclohexyl ester-ACC | Asp(OcHx) | 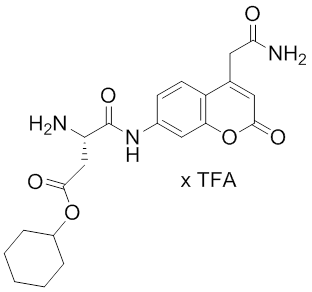 | 415,0 |
| 81 | 3,4-difluoro-L-Phenylalanine-ACC | Phe(3,4-F_2_) | 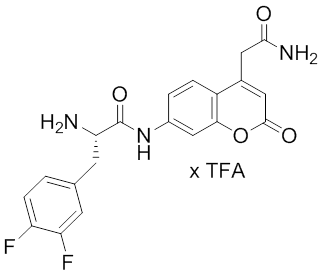 | 401,1 |
| 82 | *O*-acetyl-L-Serine-ACC | Ser(Ac) | 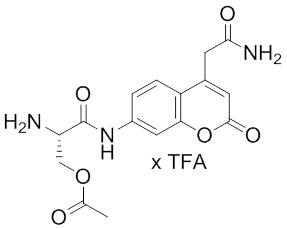 | 347,3 |
| 83 | L-Glutamic acid methyl ester-ACC | Glu(OMe) | 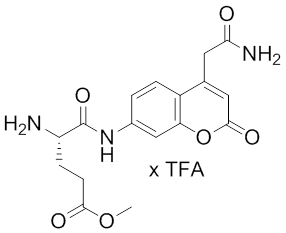 | 361,4 |
| 84 | S-4-methoxybenzyl-L-Cysteine-ACC | Cys(4-MeOBzl) | 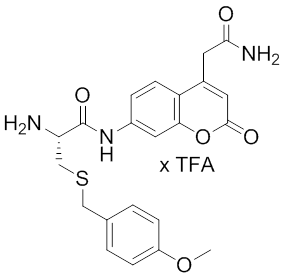 | 441,5 |
| 85 | L-thioProline-ACC | Thz | 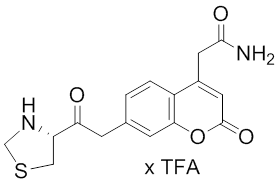 | 332,4 |
| 86 | S-4-methylbenzyl-L-Cysteine-ACC | Cys(MeBzl) | 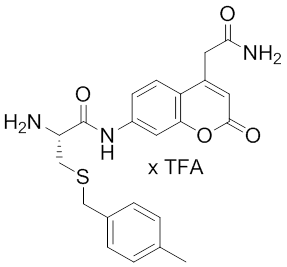 | 425,1 |
| 87 | N,N-dimethyl-L-Lysine-ACC | Lys(Me)_2_ | 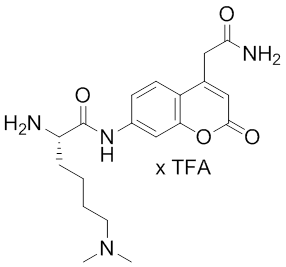 | 374,4 |
| 88 | *O*-benzyl-L-Serine-ACC | Ser(Bzl) | 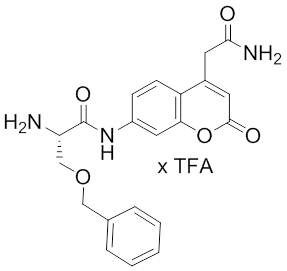 | 395,2 |
| 89 | L-Methionine sulfone-ACC | Met(O)_2_ | 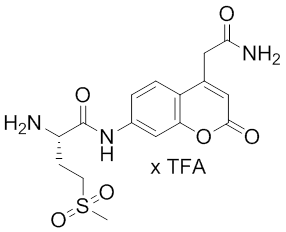 | 381,1 |
| 90 | 1-methyl-L-Tryptophan-ACC | Trp(Me) | 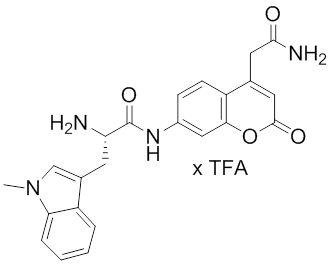 | 418,6 |
| 91 | 2-chloro-L-Phenylalanine-ACC | Phe(2-Cl) | 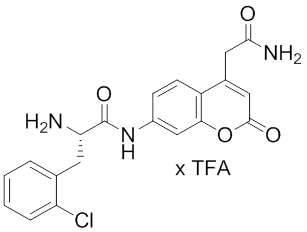 | 399,8 |
| 92 | acetyl-L-Lysine-ACC | Lys(Ac) | 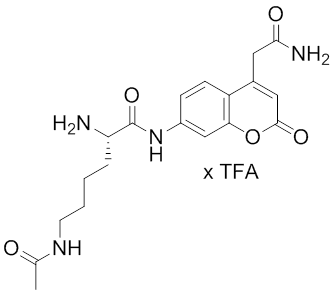 | 388,2 |
| 93 | N-6-Trifluoroacetyl-L-Lysine-ACC | Lys(Tfa) | 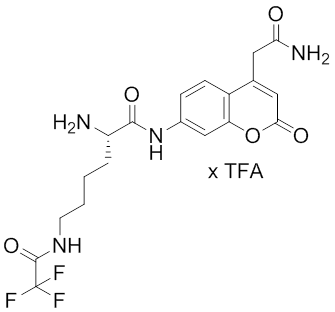 | 442,2 |
| 94 | 3-fluoro-L-Phenylalanine-ACC | Phe3F | 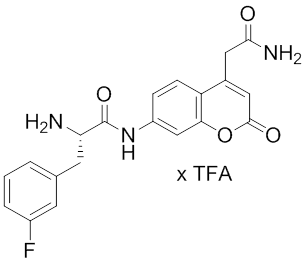 | 383,4 |
| 95 | 3,4-dichloro-L-Phenylalanine-ACC | Phe-3,4 Cl | 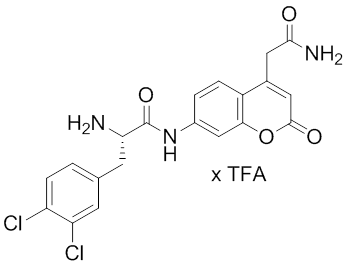 | 434,3 |
| 96 | 2-fluoro-L-Phenylalanine-ACC | Phe-2F | 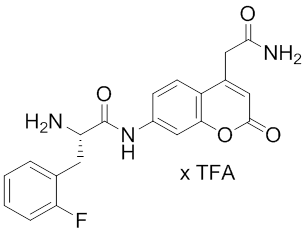 | 383,1 |
| 97 | *O*-benzyl-L-hydroxyProline-ACC | Hyp(Bzl) | 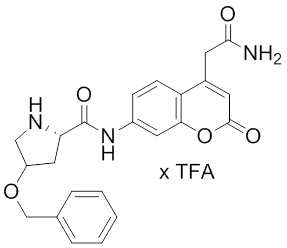 | 421,2 |
| 98 | 3-chloro-L-Phenylalanine-ACC | Phe-3Cl | 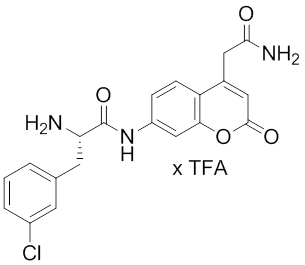 | 399,1 |
| 99 | Indoline-2-carboxylic acid-ACC | Idc | 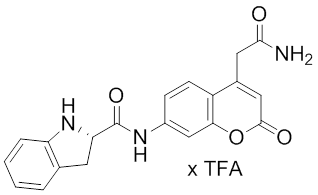 | 363,4 |
| 100 | L-octa hydroindole carboxylic acid-ACC | Oic | 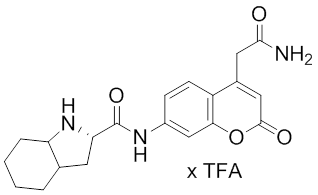 | 369,2 |
| 101 | L-homoSerine-ACC | Hse | 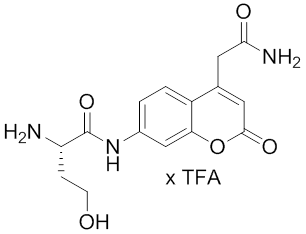 | 319,1 |
| 102 | 6-benzyloxy-L-norLeucine-ACC | Nle-6-OBzl | 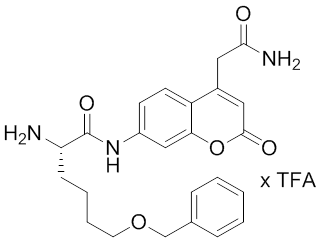 | 437,2 |
| 103 | 3-benzothiazol-2yl-L-aminobutyric acid-ACC | Abu Bht |  | 436,1 |
| 104 | nitro-L-Arginine-ACC | Arg NO_2_ |  | 419,2 |
| 105 | *O*-benzyl-L-homoSerine-ACC | HseBzl |  | 409,2 |
| 106 | L-homo norValine-ACC | Hnv |  | 331,2 |
| 107 | 4-fluoro-L-Phenylalanine-ACC | Phe-4F |  | 383,1 |
| 108 | 2,3,4,5,6-pentafluoro-L-Phenylalanine-ACC | Phe-5F |  | 455,1 |
| 109 | L-dihydroTryptophan-ACC | Dht |  | 406,2 |
| 110 | L-Aspartic acid beta-benzyl ester-ACC | Asp Bzl |  | 423,1 |
| 111 | (3-Pyridyl)-L-Alanine-ACC | 3Pal |  | 366,1 |
| 112 | D-Alanine-ACC | D-Ala |  | 289,1 |
| 113 | D-Arginine-ACC | D-Arg |  | 376,2 |
| 114 | D-Aspartic acid-ACC | D-Asp |  | 333,1 |
| 115 | D-Asparagine-ACC | D-Asn |  | 332,1 |
| 116 | D-Glutamic acid-ACC | D-Glu |  | 347,1 |
| 117 | D-Glutamine-ACC | D-Gln |  | 346,2 |
| 118 | D-Histidine-ACC | D-His |  | 355,2 |
| 119 | D-Leucine-ACC | D-Leu |  | 331,2 |
| 120 | D-Lysine-ACC | D-Lys |  | 346,2 |
| 121 | D-Methionine-ACC | D-Met |  | 349,1 |
| 122 | D-Phenylalanine-ACC | D-Phe |  | 365,2 |
| 123 | D-Proline-ACC | D-Pro |  | 315,1 |
| 124 | D-Serine-ACC | D-Ser |  | 305,1 |
| 125 | D-Threonine-ACC | D-Thr |  | 319,1 |
| 126 | D-Trypthophan-ACC | D-Trp |  | 404,2 |
| 127 | D-Tyrosine-ACC | D-Tyr |  | 381,2 |
| 128 | D-Valine-ACC | D-Val |  | 317,2 |
| 129 | D-homoPhenylalanine-ACC | D-hPhe |  | 379,1 |
| 130 | D-Phenylglycine-ACC | D-Phg |  | 351,1 |

**Table ESP2**. Mass spectrometry results for all new fluorogenic substrates used in the studies recorded in (M+H)+ mode. Data with * were recorded in (M-H)- in negative ionisation mode.

| **Name** | **Structure** | **Theoretical mass** | **MS** |
| --- | --- | --- | --- |
| (3-benzothienyl)-Ala |  | 421,4 | 422,3 |
| Met(O)OH |  | 365,4 | 366,3 |
| His(3-Bom) |  | 476,5 | 476,20 |
| Cys(Bzl) |  | 411,17 | 412,2 |
| hCyt |  | 389,41 | 390,4 |
| TyrMe |  | 395,41 | 396,30 |
| Aad |  | 361,35 | 362,3 |
| Hyp |  | 331,32 | 332,1 |
| Lys(2-Cl-Z)-OH |  | 514,96 | 515,20 |
| Glu(OcHx) |  | 429,13 | 430,20 |
| Asp(OcHx) |  | 414,99 | 416,30 |
| Phe(3,4-F_2_)-OH |  | 401,12 | 402,20 |
| Ser(Ac) |  | 347,3 | 346,1* |
| Glu(OMe) |  | 361,35 | 362,1 |
| Cys(4-MeOBzl) |  | 441,5 | 442,3 |
| Thz |  | 332,37 | 334,10 |
| Cys(MeBzl) |  | 425,14 | 426,3 |
| Lys(Me)_2_ |  | 374,43 | 375,7 |
| Ser(Bzl) |  | 395,15 | 394,3* |
| Met(O)2 |  | 381,1 | 382,2 |
| Trp(Me) |  | 418,6 | 419,20 |
| Phe (2-Cl) |  | 399,83 | 400,2 |
| Lys (Ac) |  | 388,17 | 387,2* |
| Lys (Tfa) |  | 442 | 443,20 |
| Phe3F |  | 383,37 | 384,30 |
| Phe 3,4 Cl |  | 434,27 | 434,10 |
| Phe-2F |  | 383,13 | 384,30 |
| Hyp(Bzl) |  | 421,16 | 422,40 |
| Phe 3-Cl |  | 399,1 | 400,20 |
| Idc |  | 363,37 | 364,20 |
| Oic |  | 369,17 | 370,40 |
| Hse |  | 319,3 | 320,123 |
| Nle-6-OBzl |  | 437,5 | 438,203 |
| Arg NO_2_ |  | 419,2 | 420,163 |
| Hse Bzl |  | 409,4 | 410,17 |
| Phe 4F |  | 383,4 | 384,134 |
| Phe 5F |  | 455,3 | 456,097 |
| Asp Bzl |  | 423,14 | 424,15 |
| L-3Pal |  | 366,4 | 367,14 |
| Tyr(Bzl) |  | 471,18 | 472,19 |
| Pip |  | 329,14 | 330,11 |
| Cit |  | 375,15 | 376,16 |
| Glu-benzyl ester |  | 437,16 | 438,17 |
| Tle |  | 331,15 | 332,13 |
| Dht |  | 406,13 | 407,17 |
